# Quercetin Reduces Ehrlich Tumor-Induced Cancer Pain in Mice

**DOI:** 10.1155/2015/285708

**Published:** 2015-08-13

**Authors:** Cassia Calixto-Campos, Mab P. Corrêa, Thacyana T. Carvalho, Ana C. Zarpelon, Miriam S. N. Hohmann, Ana C. Rossaneis, Leticia Coelho-Silva, Wander R. Pavanelli, Phileno Pinge-Filho, Jefferson Crespigio, Catia C. F. Bernardy, Rubia Casagrande, Waldiceu A. Verri

**Affiliations:** ^1^Department of Pathology, Biological Sciences Centre, Londrina State University, Rodovia Celso Garcia Cid KM480 PR445, Caixa Postal 10.011, 86057-970 Londrina, PR, Brazil; ^2^Department of Nursing, Health Science Centre, Londrina State University, Avenue Robert Koch 60, 86038-350 Londrina, PR, Brazil; ^3^Department of Pharmaceutical Sciences, Health Science Centre, Londrina State University, Avenue Robert Koch 60, 86038-350 Londrina, PR, Brazil

## Abstract

Cancer pain directly affects the patient's quality of life. We have previously demonstrated that the subcutaneous administration of the mammary adenocarcinoma known as Ehrlich tumor induces pain in mice. Several studies have shown that the flavonoid quercetin presents important biological effects, including anti-inflammatory, antioxidant, analgesic, and antitumor activity. Therefore, the analgesic effect and mechanisms of quercetin were evaluated in Ehrlich tumor-induced cancer pain in mice. Intraperitoneal (i.p.) treatments with quercetin reduced Ehrlich tumor-induced mechanical and thermal hyperalgesia, but not paw thickness or histological alterations, indicating an analgesic effect without affecting tumor growth. Regarding the analgesic mechanisms of quercetin, it inhibited the production of hyperalgesic cytokines IL-1*β* and TNF*α* and decreased neutrophil recruitment (myeloperoxidase activity) and oxidative stress. Naloxone (opioid receptor antagonist) inhibited quercetin analgesia without interfering with neutrophil recruitment, cytokine production, and oxidative stress. Importantly, cotreatment with morphine and quercetin at doses that were ineffective as single treatment reduced the nociceptive responses. Concluding, quercetin reduces the Ehrlich tumor-induced cancer pain by reducing the production of hyperalgesic cytokines, neutrophil recruitment, and oxidative stress as well as by activating an opioid-dependent analgesic pathway and potentiation of morphine analgesia. Thus, quercetin treatment seems a suitable therapeutic approach for cancer pain that merits further investigation.

## 1. Introduction

Approximately 50% of all cancer patients have pain [1] in early-state cancer or advanced cancer [1–4]. Cancer patients may present hyperalgesia, allodynia, and spontaneous pain, which account for poor life quality [5]. Cancer pain is a severe clinical health problem for these patients and currently the treatment for this pain is inadequate enhancing this problem [6]. In fact, at least half patients with cancer pain have received inadequate analgesic therapy [7]. One explanation for inadequate analgesic prescription could be a failure to identify pain mechanisms [2].

Several studies have demonstrated the participation of varied pathways and mediators involved in cancer pain development, such as cytokines [8–10], spinal glial activation [11–14], transient receptor potential vanilloid receptor 1 (TRPV1), acid-sensing ion channels (ASICs), bradykinin, adenosine triphosphate (ATP), endothelin [15], reactive oxygen species [16], and intracellular signaling pathway such as mitogen-activated protein kinases p38 [17] and JNK [18]. Cancer pain mechanisms are also dependent on the cancer type implicating that some slight variations in the mechanisms or role of a certain pathway may be greater depending on cancer type. Therefore, cancer pain is a complex condition and as already mentioned its control might also depend on adequate pharmacological tools. Opioids are effective clinically used analgesics in cancer pain; however, they have many side effects that increase with the dose of opioid and, in addition to tolerance, the dose regimen increases with the tumor growth [19]. Thus, it is important to find novel therapeutic approaches to reduce cancer pain and/or improve current clinical therapies.

Flavonoids such as quercetin present low toxicity [20], which together with its antinociceptive effect in models of inflammation [21] and neuropathic pain [22] suggests its usefulness as an analgesic drug. Moreover, cancer pain might present components of inflammatory pain related to the inflammatory response against the tumor cells and neuropathic pain related to neuronal damage and nerve compression. It has been demonstrated in models of inflammation that the mechanisms of quercetin are related to inhibition of oxidative stress and cytokine production [23, 24]. In models of diabetic neuropathic pain, quercetin induces an analgesic effect amenable by opioid receptor antagonist [22]. In fact, inhibition of oxidative stress, cytokine production, and opioid receptor-dependent effects seem to be major mechanisms of quercetin since they were also observed in models such as colitis [25], neuropathy [26], hepatic fibrosis [27], periodontitis-induced bone resorption [28], and allergic inflammation [29].

In the present study, the analgesic activity and mechanisms of quercetin were investigated in Ehrlich tumor-induced cancer pain in mice [30]. This is a model of murine mammary adenocarcinoma-induced pain presenting features like those of preoperative breast cancer with spontaneous pain and pain upon examination (pressure of the lump, hyperalgesia) [30–32] with the benefit of development in standard Swiss mice. Furthermore, Ehrlich tumor induces bone/cartilage destruction indicating the possible involvement of a bone pain component in its nociceptive mechanisms [30].

## 2. Material and Methods

### 2.1. General Experimental Procedures

The measurement of basal responses to mechanical and thermal stimuli and paw thickness was performed at day 0. Afterwards, mice received intraplantar (i.pl.) injection of Ehrlich tumor cells (1 × 10^6^ or 1 × 10^7^). Ehrlich's tumor cells are cultivated* in vivo*, by passages in the peritoneum of Swiss mice in ascitic form. Ten days after the intraperitoneal (i.p.) injection of 0.2 mL of ascitic peritoneal fluid containing Ehrlich tumor cells in mice, the ascitic fluid of tumor cells was collected and washed in phosphate-buffered saline (PBS, pH 7.4) followed by centrifugation (200 g, 10 min) three times. The cell viability was determined by 0.5% trypan blue exclusion method in Neubauer chamber. Ehrlich tumor cells were suspended to the final concentrations of 1 × 10^6^ or 1 × 10^7^ in 25 *μ*L of saline and injected into the subcutaneous tissue of mice, which passes from ascitic form to solid form [30]. Mice received the Ehrlich tumor cells (1 × 10^6^ or 1 × 10^7^ in 25 *μ*L of saline) and received the acute treatment with quercetin (10–100 mg/kg, i.p.) or vehicle (2% DMSO in saline) on the 8th day after injection of the cells, and mechanical and thermal hyperalgesia and paw thickness were determined after 1, 3, 5, and 7 h. For chronic treatment, mice were treated with quercetin (10–100 mg/kg, i.p) 10 min after Ehrlich tumor cells injection followed by daily treatment. Mechanical and thermal hyperalgesia and paw thickness were evaluated on days 2, 4, 6, 8, 10, and 12 after the injection of 1 × 10^6^ cells and 3 h after treatment with quercetin. A control group received saline (25 *μ*L/paw, vehicle of Ehrlich tumor cells) and quercetin (100 mg/kg, i.p) treatment. On the 12th day of the injection of tumor cells, 3 h after the daily treatment with quercetin (100 mg/kg i.p., both tumor and saline group) or vehicle, paw samples were collected for histological analysis and microscopic observation. Paw skin and spinal cord samples were collected to determine myeloperoxidase (MPO) activity, interleukin-1*β* (IL-1*β*), and tumor necrosis factor *α* (TNF*α*) concentration by ELISA, FRAP, ABTS, and GSH levels. In another set of experiments, mice received 1 × 10^7^ Ehrlich tumor cells or saline and were treated with quercetin (100 mg/kg, i.p.) or vehicle starting 10 min after Ehrlich tumor cells injection and followed by daily treatment during 8 days. On the 8th day, 3 h after treatment, the overt pain-like behavior was assessed. In other experiments, mice received Ehrlich tumor cells (1 × 10^6^ or 1 × 10^7^) and were treated with quercetin (100 mg/kg, i.p.) or vehicle daily during 8 days; on the 8th day, mice received the treatment with naloxone (1 mg/kg i.p.) (an opioid receptor antagonist) followed by evaluation of mechanical and thermal hyperalgesia, paw thickness, overt pain, and collection of spinal cord and paw skin and samples for evaluation of myeloperoxidase (MPO) activity (only paw skin), IL-1*β* and TNF*α* concentration, FRAP, ABTS, and GSH levels. Lastly, we assessed the effect of cotreatment with quercetin (10 mg/kg, i.p.) and morphine (1 mg/kg, i.p.) (at doses that were not effectively analgesic as single treatment) over Ehrlich tumor-induced (1 × 10^6^ or 1 × 10^7^ cells) mechanical hyperalgesia, thermal hyperalgesia, paw thickness, and overt pain-like behavior. Time points of the analyzed parameters were standardized in our laboratory [30].

### 2.2. Test Compound

The compounds used in this study were PBS pH 7.4, saline (NaCl 0.9%, Fresenius Kabi Brasil Ltda., Aquiraz, CE, Brazil), Tween, and DMSO 2%, and quercetin at 95% purity was purchased from Acros Organics (Fair Lawn, NJ, USA).

### 2.3. Ehrlich Tumor Cells

Peritoneal ascitic fluid of mice that received Ehrlich tumor cells i.p. was collected and injected in other mice. Ten days after the injection of ascitic fluid containing Ehrlich tumor cells, the ascitic fluid was collected for experiments. Ehrlich tumor cells were developed by Paul Ehrlich in 1896 and described as a spontaneous breast adenocarcinoma of female mice. It was originally developed as an ascitic form but can be converted to solid form when inoculated into tissues. Injection of Ehrlich tumor cells in the paw induces mechanical hyperalgesia, thermal hyperalgesia, increase of paw thickness, and overt pain-like behavior [30].

### 2.4. Animals

Male Swiss mice (25–30 g), from the Universidade Estadual de Londrina, Londrina, Parana, Brazil, were used in this study. Mice were housed in standard clear plastic cages with free access to food and water and a light/dark cycle of 12 : 12 h and kept at 21°C. All behavioral testing was performed between 9 a.m. and 5 p.m. in a temperature-controlled room. Animal care and handling procedures were approved by the Ethics Committee of the Universidade Estadual de Londrina (13279.2011.76). Every effort was made to minimize the number of animals used and their suffering.

### 2.5. Mechanical Hyperalgesia

Mechanical hyperalgesia was evaluated as previously reported [30]. In a quiet room, mice were placed in acrylic cages (12 × 10 × 17 cm) with wire grid floors, 15–30 min before the start of testing. The test consisted of evoking a hindpaw flexion reflex with a hand-held force transducer (electronic anesthesiometer, Insight, Ribeirão Preto, SP, Brazil) adapted with a 0.5 mm^2^ polypropylene tip. The investigator was trained to apply the tip perpendicularly to the central area of the hindpaw with a gradual increase in pressure. The end point was characterized by the removal of the paw followed by clear flinching movements. After the paw withdrawal, the intensity of the pressure was recorded automatically. The value for the response was an average of three measurements. The animals were tested before and after treatment. The results are expressed by delta (Δ) withdrawal threshold (in g) calculated by subtracting the mean measurements at 1, 3, 5, and 7 h after acute treatment on the 8th day after injection of the Ehrlich tumor cells or 3 h after each daily treatment with quercetin in the chronic protocol on days 2, 4, 6, 8, 10, and 12 after injection of the cells from the zero-time mean measurements.

### 2.6. Thermal Hyperalgesia

Mice were placed in a 10 cm-wide glass cylinder on a hot plate (IITC Life Science, Inc., Woodland Hills, CA, United States) maintained at 55°C. Two control latencies of at least 10 min apart were determined for each mouse. The normal latency (reaction time) was 10–15 s. The reaction time was scored when the animal jumped or licked its paws. A maximum latency (cut-off) was set at 20 s to avoid tissue damage [30]. The results are expressed as thermal threshold.

### 2.7. Paw Thickness or Tumor Growth

Paw thickness was determined before and at indicated time points (at 48 h intervals) after the injection of Ehrlich tumor cells using an analog caliper. Paw thickness/tumor growth was presented as Δ mm [30].

### 2.8. Overt Pain-Like Behavior Evaluation

Mice received 1 × 10^7^ cells/paw in 25 *μ*L and were placed in clear glass compartments at room temperature. After an acclimation period of 10 min, mice were observed during 10 min, and the cumulative number of flinches was determined [30].

### 2.9. Histopathological Analyses

Twelve days after the injection of the Ehrlich tumor cells, mice were euthanized and the paw was removed and decalcified in EDTA solution during 35 days. Samples were embedded in paraffin, sectioned into 5 *μ*m section, and stained with hematoxylin and eosin for light microscopic analysis [30].

### 2.10. Myeloperoxidase (MPO) Activity

Neutrophil recruitment to the paw skin was evaluated by the MPO kinetic-colorimetric assay [25]. Paw skin samples were collected in 50 mM K_2_PO_4_ buffer (pH 6.0) containing 0.5% HTAB and were homogenized using a Polytron (PT3100). After the homogenates were centrifuged at 16.100 g for 2 min, the resulting supernatant was assayed for MPO activity at 450 nm (Multiskan GO Microplate Spectrophotometer) with three readings within 1 min. The MPO activity of the samples was compared with a standard curve of neutrophils. The results were presented as the MPO activity (number of neutrophils × 10^5^/mg of tissue).

### 2.11. Cytokine Measurement

Mice spinal cord (L4–L6) and paw skin samples were collected and homogenized in 500 *μ*L of buffer containing protease inhibitors, and IL-1*β* and TNF*α* levels were determined as described previously by an enzyme-linked immunosorbent assay (ELISA) using eBioscience kits. The results were expressed as picograms (pg) of cytokine/mg of spinal cord or paw skin. As a control, the concentrations of these cytokines were determined in animals injected with saline and treated with vehicle [25].

### 2.12. Antioxidants Tests

Spinal cord and paw skin tissue samples were collected and immediately homogenized with 500 *μ*L of 1.15% KCl. Samples were centrifuged (10 min, 0.2 g, and 4°C) and the total antioxidant capacity was determined by the FRAP (ferric reducing ability potential) and ABTS (ability to scavenge ABTS radical) assays [25]. For FRAP assay, 50 *μ*L of supernatant was mixed with 150 *μ*L of deionized water and 1.5 mL of FRAP reagent freshly prepared. The reaction mixture was incubated at 37°C for 30 min and absorbance was measured at 595 nm. For ABTS assay, ABTS solution was diluted with phosphate buffer saline pH 7.4 (PBS) to an absorbance of 0.80 at 730 nm. Then, 1.0 mL of diluted ABTS solution was mixed with 20 *μ*L of supernatant. After 6 min, the absorbance was measured at 730 nm. The results were equated against a Trolox standard curve (1.5–30 *μ*mol/L, final concentrations). The results were expressed as Trolox equivalents per gram of spinal cord or paw skin in both assays. For GSH measurement, spinal cord and paw skin samples were collected and maintained at −80°C for at least 48 h. Samples were homogenized with 200 *μ*L of 0.02 M EDTA. The homogenate was mixed with 25 *μ*L of 50% trichloroacetic acid and was homogenized three times during 15 min. The mixture was centrifuged (15 min, 1.5 g, and 4°C). The supernatant was added to 200 *μ*L of 0.2 M TRIS buffer, pH 8.2, and 10 *μ*L of 0.01 M DTNB. After 5 min, the absorbance was measured at 412 nm against a reagent blank with no supernatant. A standard curve with GSH was performed. The results are expressed as GSH per mg of protein of spinal cord or paw skin [25]. For protein determination, 60 *μ*L of supernatant was mixed with 60 *μ*L of copper reagent freshly prepared. After 10 min, 180 *μ*L of Folin solution was added. The resulting solution was incubated at 50°C for 10 min. The absorbance was measured at 660 nm and the results equated to a standard curve of bovine serum albumin [33].

### 2.13. Statistical Analysis

Results are presented as means ± SEM of measurements made on six mice in each group per experiment and are representative of two independent experiments. Two-way analysis of variance (ANOVA) was used to compare the groups and doses at all times (curves) when the hyperalgesic responses were measured at different times after the administration or enforcement of the stimuli. The factors analyzed were treatment, time, and time versus treatment interaction. When there was a significant time versus treatment interaction, one-way ANOVA followed by Tukey's *t*-test was performed on each occasion. Statistical differences were considered to be significant at *p* < 0.05.

## 3. Results and Discussion

### 3.1. Quercetin Inhibits Pain-Like Behavior and Neutrophil Recruitment Induced by Ehrlich Tumor Cells

**Figure 1:**
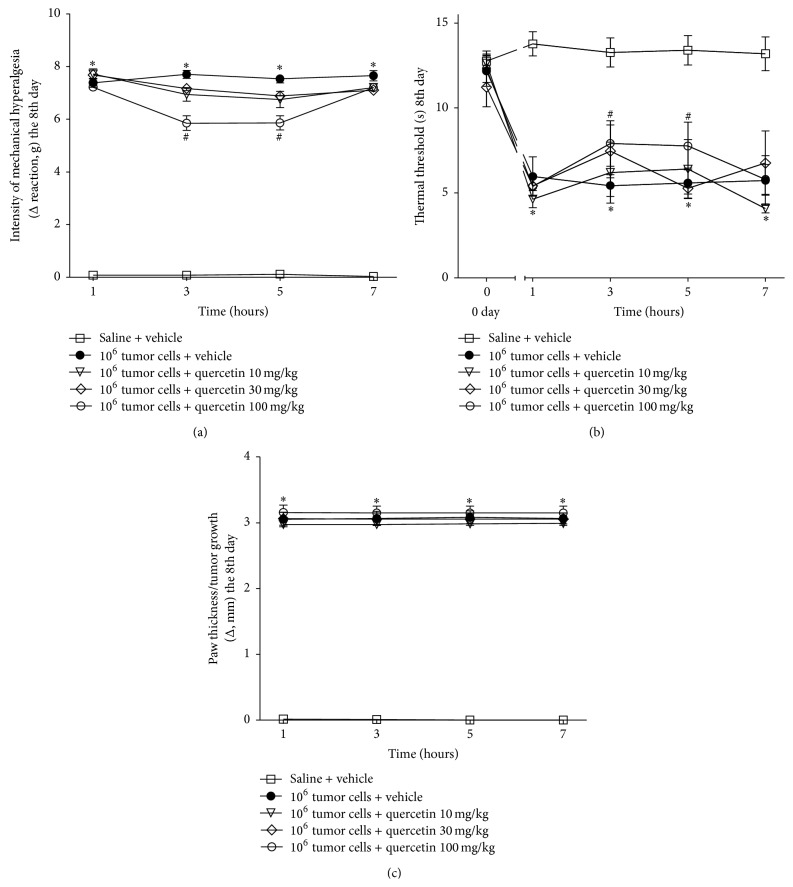
Acute treatment with quercetin inhibits Ehrlich tumor-induced pain-like behavior in mice. Mice received the intraplantar (i.pl.) administration of Ehrlich tumor cells 1 × 10^6^ (a–c), and in the 8th day after injection, the tumor cells mice received the acute treatment with quercetin (10, 30, and 100 mg/kg i.p.). Mechanical hyperalgesia (a), thermal hyperalgesia (b), and paw thickness (c) were accessed at 1, 3, 5, and 7 hours after the treatment. Data are presented as means ± SEM of six mice per group per experiment and are representative of two separated experiments: ^*∗*^ *p* < 0.05 compared to the saline group and ^#^ *p* < 0.05 compared to the tumor group. One-way ANOVA followed by Tukey's test.

**Figure 2:**
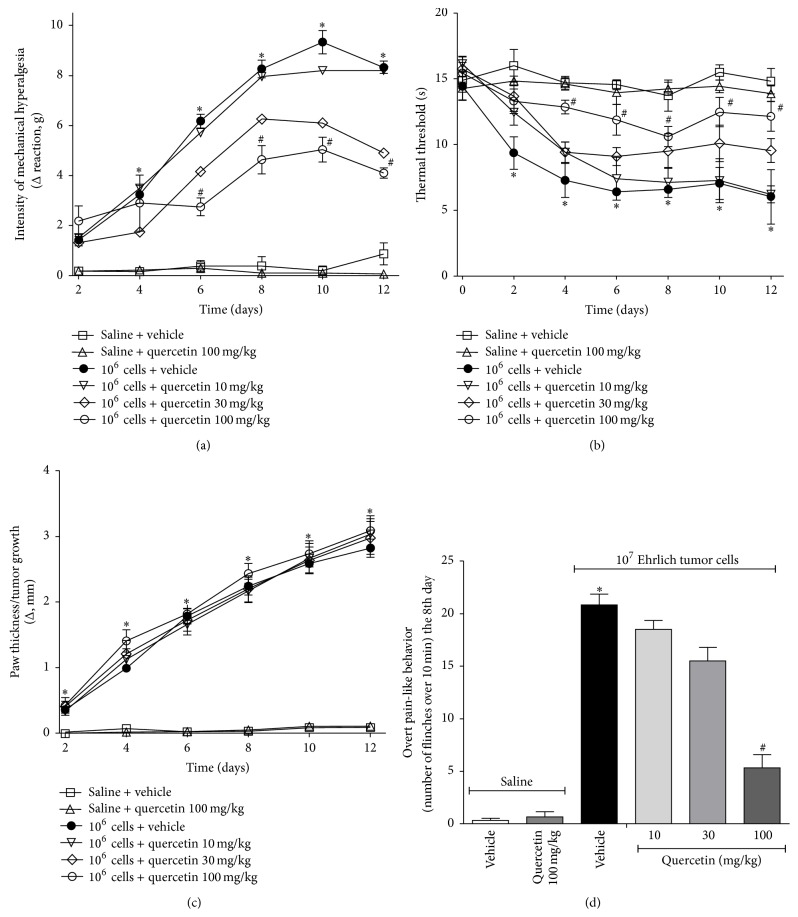
The chronic treatment with quercetin inhibits in a dose-dependent manner Ehrlich tumor-induced pain-like behavior in mice. Mice received the intraplantar (i.pl.) administration of Ehrlich tumor cells (1 × 10^6^ (a–c) or 1 × 10^7^ (d)) and were treated daily with quercetin (10, 30, and 100 mg/kg i.p.) during 12 days (a–c) or 8 days (d) starting 10 min after tumor injection. The control group of Ehrlich tumor vehicle was saline and saline plus quercetin group was a control of possible* per se* effects of quercetin. Mechanical hyperalgesia (a), thermal hyperalgesia (b), paw thickness (c), and overt pain-like behavior (d) were evaluated 3 h after the treatment. Data are presented as means ± SEM of six mice per group per experiment and representative of two separated experiments: ^*∗*^ *p* < 0.05  compared to the saline group and ^#^ *p* < 0.05 compared to the tumor group. One-way ANOVA followed by Tukey's test.

Ehrlich tumor cells induced significant mechanical hyperalgesia starting at the 4th day up to the 12th day and thermal hyperalgesia starting at the 2nd day up to the 12th day confirming previous standardization [30]. The acute analgesic effect of quercetin (10–100 mg/kg, i.p. 2% DMSO diluted in saline) was assessed on the 8th day after injection of the Ehrlich tumor cells at 1, 3, 5, and 7 h after treatment. Quercetin (100 mg/kg, i.p.) treatment significantly reduced the mechanical and thermal hyperalgesia at 3 and 5 h after treatment (Figures 1(a) and 1(b), resp.) but did not alter the paw thickness (Figure 1(c)). The chronic posttreatment with quercetin (10–100 mg/kg, 2% DMSO diluted in saline) significantly reduced the mechanical hyperalgesia from days 6 to 12 (Figure 2(a)) and thermal hyperalgesia between 4 and 12 days (Figure 2(b)) in a dose-dependent manner. The inhibition of Ehrlich tumor-induced mechanical and thermal hyperalgesia was not accompanied by alteration of paw thickness, indicating that quercetin did not affect tumor growth (Figure 2(c)). The treatment with quercetin (100 mg/kg, i.p) of mice that received Ehrlich tumor vehicle (saline) did not alter the basal mechanical or thermal hyperalgesia, or paw thickness (Figures 2(a)–2(c)) indicating that quercetin did not present* per se* effects.

In the present model, Ehrlich tumor cells induced overt pain-like behavior, such as paw flinching, at the dose of 1 × 10^7^ cells with peak of response at the 8th day after injection [30]. At this time point, the daily treatment with quercetin also inhibited Ehrlich tumor-induced paw flinching (Figure 2(d)) with significant analgesic effect with the dose of 100 mg/kg of quercetin over 10 and 30 mg/kg. There was no effect on mice that received Ehrlich tumor vehicle (saline) plus quercetin treatment (100 mg/kg, i.p.). Considering the results of Figures 1 and 2, the dose of 100 mg/kg of quercetin was selected for the next experiments. Corroborating the present data, quercetin also inhibited mechanical hyperalgesia, thermal hyperalgesia, and overt pain-like behavior induced by varied stimuli in other models of inflammatory and neuropathic pain [21, 22, 34, 35], and the dose of 100 mg/kg of quercetin was also selected [21, 22, 25, 36].

**Figure 3:**
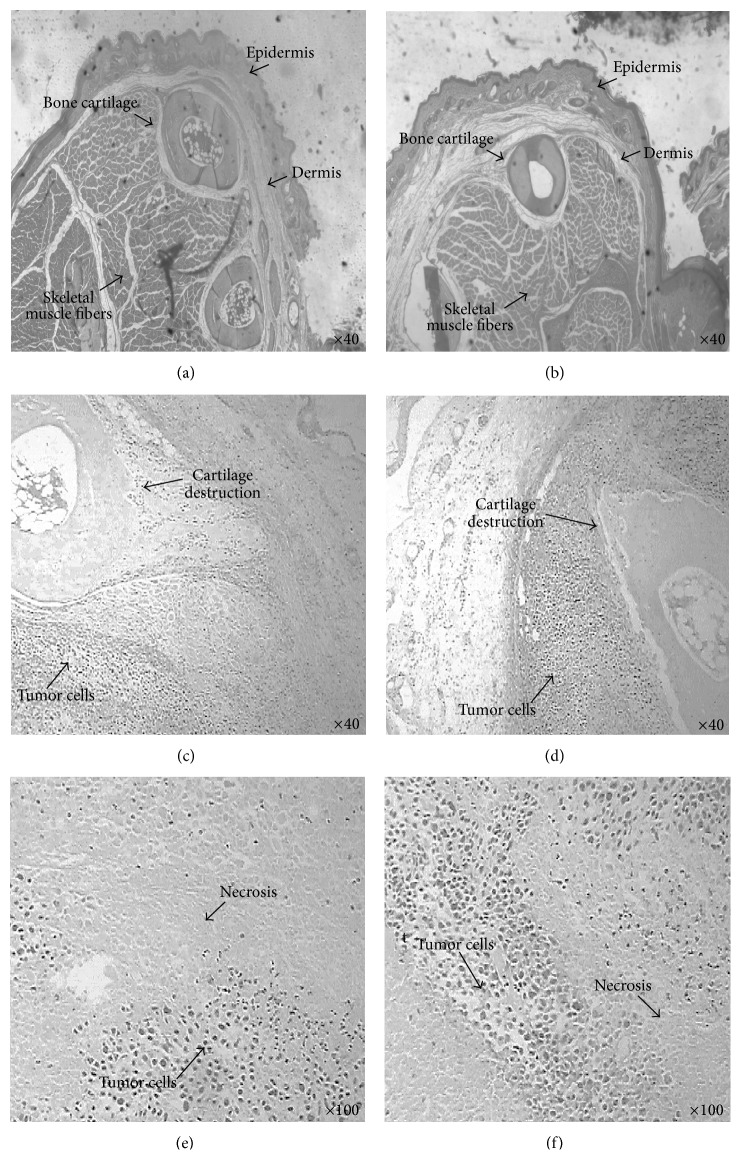
Quercetin does not alter Ehrlich tumor-induced histological modifications. Mice received saline (25 *μ*L) or Ehrlich tumor cells (1 × 10^6^/25 *μ*L) and were treated i.p. with quercetin (100 mg/kg, 2% DMSO diluted in saline) or vehicle (2% DMSO) 10 min after the i.pl. injection. The treatment continued daily during 12 days. In the 12th day, mice were euthanized and the paw was collected for histological analysis performed with hematoxylin/eosin staining. Panel (a) shows the histology of saline i.pl. plus quercetin vehicle group, (b) saline i.pl. plus quercetin (100 mg/kg i.p.), (c and e) tumor animal treated with vehicle, and (d and f) tumor animal treated with quercetin (100 mg/kg i.p.). Arrows indicate intact bone cartilage, presence of skeletal muscle fibers, dermis and epidermis: (a-b) bone/cartilage destruction (c-d), tissue necrosis (e-f), and presence of tumor cells (c–f).

In agreement with the results of Figure 2(c), hematoxylin/eosin staining of paw samples revealed no histological differences between mice with tumor treated with quercetin and vehicle control group. Mice that received saline in the paw and were treated with the vehicle of quercetin (Figure 3(a)) or quercetin (100 mg/kg i.p.) (Figure 3(b)) showed normal tissue. The arrows show the presence of epidermis, dermis, skeletal muscle fibers, and intact bone and cartilage. On the other hand, mice that received Ehrlich tumor cells and were treated with the vehicle of quercetin (Figures 3(c) and 3(e)) or with quercetin (100 mg/kg i.p) (Figures 3(d) and 3(f)) presented cartilage destruction, tissue necrosis, and intense tumor proliferation. This could be seen as a drawback data in the sense that quercetin does not inhibit Ehrlich tumor cells growth and, therefore, quercetin does not present an antitumor effect at this analgesic dose. On the other hand, the positive side is that quercetin exerts an analgesic effect without affecting tumor growth; thus, it is suitable for treatment of cancer pain and does not promote tumor growth. Nevertheless, some studies have shown the antitumor effect of quercetin. For instance, treatment with quercetin induced apoptosis and/or inhibited the growth of human breast carcinoma MCF-7 cells [37], K562 human chronic myeloid leukemia, Molt-4 acute T-lymphocytic leukemia, Raji Burkitt lymphoma [38], nasopharyngeal carcinoma cells [39], and other cancer cell lines [40, 41]. Dose of treatment,* in vivo* versus* in vitro* contexts, and cancer cell lines are some possible explanations for this divergent data. Nevertheless, it is possible that higher doses of quercetin would present antitumor effect with improved analgesia since it would present an intrinsic analgesic effect plus reduction of tumor and the immune response against the tumor.

**Figure 4:**
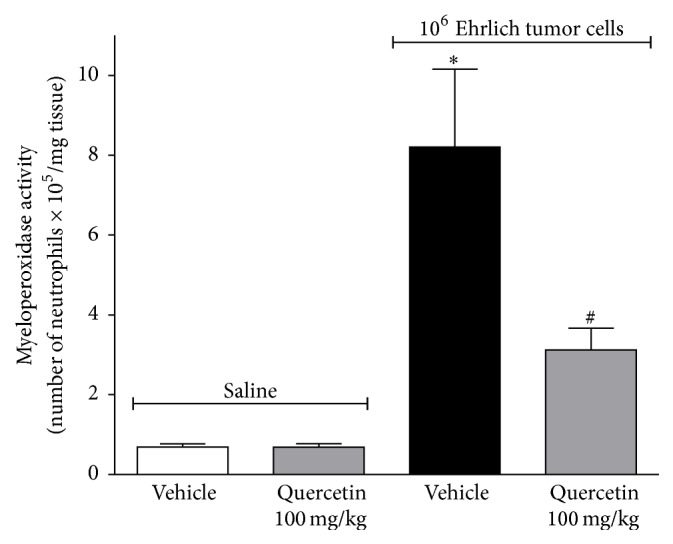
Quercetin inhibits neutrophil recruitment induced by Ehrlich tumor cells. Mice were treated i.p. with quercetin (100 mg/kg, 2% DMSO diluted in saline) or vehicle (2% DMSO) 10 min after the injection of Ehrlich tumor cell (1 × 10^6^/25 *μ*L) or saline (25 *μ*L). The neutrophil recruitment was evaluated in samples of paw skin collected after 12 days of treatment using the myeloperoxidase (MPO) activity assay. Data are presented as means ± SEM of six mice per group per experiment and representative of two separated experiments: ^*∗*^ *p* < 0.05 compared to the saline group and ^#^ *p* < 0.05 compared to the tumor group. One-way ANOVA followed by Tukey's test.

There is evidence that ascitic Ehrlich tumor induces the recruitment to the peritoneal cavity of mice of cellular populations consistent with dendritic cells, monocytes, and neutrophils [42]. In the present study, it was observed that Ehrlich tumor injection in the paw induces an increase of myeloperoxidase (MPO) activity and daily treatment with quercetin (100 mg/kg, i.p.) inhibited this increase of MPO activity (Figure 4). The saline group treated with quercetin (100 mg/kg, i.p) did not present alteration of MPO activity compared to quercetin vehicle. MPO is an important enzyme of neutrophil microbicidal activity and is used as a marker of inflammation and neutrophil recruitment [43]. The inhibition of neutrophil recruitment or activation is an analgesic mechanism since recruited neutrophils contribute to hyperalgesia by further producing nociceptive molecules [43]. Therefore, inhibiting neutrophil recruitment might be accounting for the analgesic effect of quercetin. In addition to neutrophils, macrophages express MPO, suggesting that the inhibition of MPO activity by quercetin treatment could also involve the reduction of macrophage counts. This is consistent with the demonstration that Ehrlich tumor cells induce the recruitment of monocytes, which could differentiate into macrophages [42]. Treatment with quercetin also inhibits MPO activity* in vitro* [44] and also the neutrophil recruitment* in vivo* and neutrophil chemotaxis* in vitro* induced by chemokines, fMLP (formyl-methionyl-leucyl-phenylalanine) and leukotriene B_4_ [40]. Therefore, quercetin is able to inhibit the MPO enzyme as well as the recruitment of cells expressing MPO. In addition to inhibiting the chemotactic effects of cytokines, peptides, and lipid mediators, quercetin also inhibits the production of such molecules. Thus, in the next set of experiments, whether quercetin would inhibit the production of cytokines with hyperalgesic and chemotactic functions such as IL-1*β* and TNF*α* was investigated [45].

### 3.2. Quercetin Inhibits IL-1*β* and TNF*α* Production Induced by Ehrlich Tumor Cells in the Spinal Cord and Paw Skin

**Figure 5:**
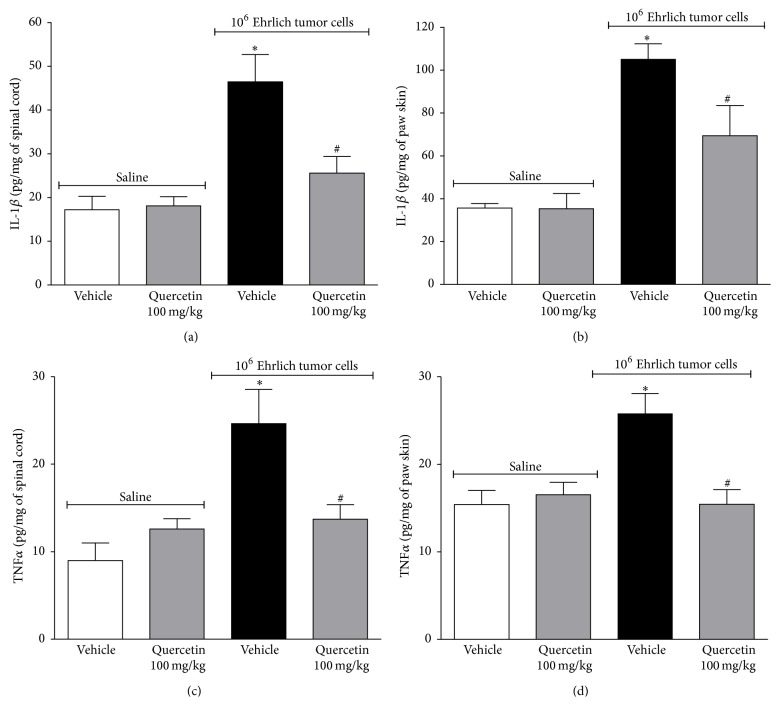
Quercetin inhibits IL-1*β* and TNF*α* production induced by Ehrlich tumor cells in the spinal cord and paw skin. Mice were treated i.p. with quercetin (100 mg/kg, 2% DMSO diluted in saline) or vehicle (2% DMSO) 10 min after the injection of Ehrlich tumor cell (1 × 10^6^/25 *μ*L) or saline (25 *μ*L). The treatment continued daily. In the 12th day after injection, the Ehrlich tumor cells, the spinal cord, and the paw skin samples were collected for cytokine measurement. IL-1*β* in spinal cord (a) or paw skin (b) and TNF*α* in spinal cord (c) or paw skin (d) were determined by ELISA. Data are presented as means ± SEM of six mice per group per experiment and representative of two separated experiments: ^*∗*^ *p* < 0.05 compared to the saline group and ^#^ *p* < 0.05 compared to the tumor group. One-way ANOVA followed by Tukey's test.

Mice received daily treatment during 12 days with quercetin (100 mg/kg, i.p.) starting 10 min after the injection of saline or Ehrlich tumor (1 × 10^6^, i.pl.) injection as described at Figure 2, and samples were collected in the 12th day (Figure 5). Ehrlich tumor cells induced significant production of IL-1*β* in the spinal cord (Figure 5(a)) and in the paw skin (Figure 5(b)). TNF*α* levels were also increased in spinal cord (Figure 5(c)) and paw skin (Figure 5(d)). Quercetin treatment inhibited Ehrlich tumor-induced IL-1*β* and TNF*α* production in the spinal cord and paw skin (Figure 5). The daily treatment with quercetin (100 mg/kg i.p.) in mice that received i.pl. control saline did not alter the production of cytokines compared to naive group. Cytokines including IL-1*β* and TNF*α* have spinal cord and peripheral nociceptive roles as observed in inflammation and neuropathic and cancer models. Therefore, inhibiting IL-1*β* and/or TNF*α* production or action at the central spinal cord or peripheral levels is a promising analgesic approach [45]. In fact, the intrathecal treatment with IL-ra (an IL-1 receptor antagonist) inhibited the hyperalgesia induced by AT-3.1 prostate cancer cells into the tibia of rats [46] and systemic treatment with anakinra (an IL-1 receptor antagonist) reduced the hyperalgesia induced by osteosarcoma model of bone cancer pain [47]. The i.pl. injection of lung carcinoma cells induces hyperalgesia in mice accompanied by high peripheral production of IL-1*β* and TNF*α*, and the treatment with etanercept (a TNF-neutralizing soluble receptor) prevented the development of heat hyperalgesia. Furthermore, TNF-induced cancer-related heat hyperalgesia through nociceptor sensitization is linked to upregulation of transient receptor potential vanilloid 1 (TRPV1) [8]. Similarly, etanercept also reduced the mechanical hyperalgesia in a bone cancer model [13, 48]. The nociception triggered by IL-1*β* and TNF*α* presents peripheral and central spinal cord mechanisms. For instance, TNF*α* triggers acute inflammatory hyperalgesia by inducing IL-1*β* production, which in turn induces prostaglandin E_2_ production. Prostaglandin E_2_ is ultimately responsible for sensitization of nociceptive neurons [45]. After the first inflammatory stimulus, there is a condition named hyperalgesic priming representing prolonged inflammation in which TNFR1 expression is induced in nociceptive neurons and, therefore, TNF*α* can exert a direct sensitizing effect [49].* In vitro*, dorsal root ganglia (DRG) neurons express TNFR1 receptors possibly due to the collection procedure of the DRG, which resembles axotomy. In this condition, TNF*α* induces p38 mitogen-activated protein (MAP) kinase activation that phosphorylates tetrodotoxin-resistant sodium channels resulting in neuronal depolarization [50]. TNFR1 and TNFR2 also participate in the spinal cord activation of astrocytes and pain [13]. In cancer, the inhibition of p38-MAPK signaling pathway attenuates breast cancer-induced bone pain [17]. TNFR2 also plays a pronounced role in lung carcinoma cells-induced heat hyperalgesia [8]. Therefore, cytokines such as IL-1*β* and TNF*α* are involved in the neuronal activation at peripheral sites, DRG, and spinal cord in varied painful conditions and targeting these cytokines is one of the efficient analgesic approaches in cancer pain.

### 3.3. Quercetin Inhibits the Oxidative Stress Induced by Ehrlich Tumor Cells

**Figure 6:**
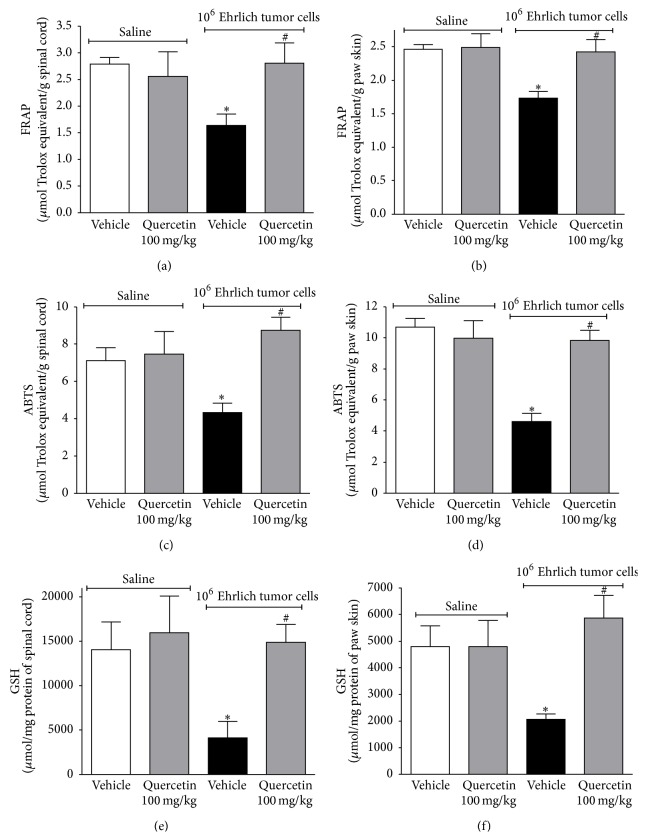
Quercetin inhibits the oxidative stress induced by Ehrlich tumor cells. Mice were treated with quercetin (100 mg/kg, i.p.) or vehicle 10 min after the injection of Ehrlich tumor cells (1 × 10^6^/25 *μ*L) or saline. The treatment continued daily during 12 days, and, in the 12th day, 3 h after the treatment, samples of spinal cord and paw skin were collected for the oxidative stress assays. The FRAP and ABTS ability of spinal cord ((a) and (c), resp.) and paw skin ((b) and (d), resp.) tissues and GSH levels in spinal cord (e) and paw skin (f) were accessed. Data are presented as means ± SEM of six mice per group per experiment and representative of two separated experiments: ^*∗*^ *p* < 0.05 compared to the saline group and ^#^ *p* < 0.05 compared to the tumor group. One-way ANOVA followed by Tukey's test.

There is close relation between cytokines and oxidative stress in pain induction. IL-1*β* and TNF*α* can activate nicotinamide adenine dinucleotide phosphate- (NADPH-) oxidase, resulting in the production of superoxide anion. In turn, superoxide anion activates nuclear factor kappa B (NF*κ*B) and enhances cytokine production [23, 51, 52]. In this sense, the effect of quercetin on Ehrlich tumor-induced oxidative stress was accessed by the total antioxidant capacity depletion in the spinal cord and paw skin using the ability to ferric reducing potential (FRAP) assay, scavenge 2,2′-azinobis-(3-ethylbenzothiazoline 6-sulfonic acid radical) (ABTS) assay, and reduced glutathione (GSH) levels. Mice were divided and treated as in Figure 2 and samples were collected in the 12th day. Ehrlich tumor cells induced oxidative stress (Figure 6). The quercetin treatment showed a significant increase in FRAP at both the spinal cord (Figure 6(a)) and paw skin (Figure 6(b)) and ABTS in the spinal cord (Figure 6(c)) and paw skin (Figure 6(d)). It is known that quercetin is an antioxidant flavonoid and its effects could be explained by the presence of structural antioxidant chemical groups [53]. However, there is no antioxidant structural relationship of flavonoids and the inhibition of intracellular signaling pathways such as mitogen-activated protein kinases [23]. Therefore, the presence of structural antioxidant chemical groups does not fully explain the activities of quercetin.

In cancer and during chemotherapy treatment, there is increased production of reactive species [54], which can result in antioxidant depletion and, consequently, oxidative stress. The main consequence of rapid cellular division in cancer is the increase of the metabolic by products, such as excessive production of reactive oxygen species (ROS) [55]. Decreased levels of GSH have been reported in patients with breast cancer [56, 57]. The increased oxidative stress gives rise to inflammation, which could further aggravates the pain [57]. In this sense, quercetin may present an important applicability in reducing cancer-induced oxidative stress. It is noteworthy that the inhibition of peripheral oxidative stress observed may also be attributed to the reduction in neutrophil recruitment by quercetin (Figure 4), because activated neutrophils are important sources of reactive oxygen and nitrogen species in the tissue. Quercetin also inhibited Ehrlich tumor-induced GSH depletion in the spinal cord (Figure 6(e)) and paw skin (Figure 6(f)). This is in agreement with previous studies demonstrating that quercetin presents beneficial effects through antioxidant activities in other experimental models such as colitis [25] and inflammatory pain [21]. It has been suggested that the prevention of GSH depletion may be an important analgesic mechanism [58]. GSH can reduce reactive species and is an important molecule of the endogenous antioxidant system. In this sense, the preservation of GSH levels by quercetin may also prevent total antioxidant capacity depletion and oxidative stress [54]. Therefore, the antinociceptive activity of quercetin could also be associated with the inhibition of oxidative stress in this model.

### 3.4. Quercetin Analgesia, but Not the Anti-Inflammatory Effect, Depends on Endogenous Opioids

**Figure 7:**
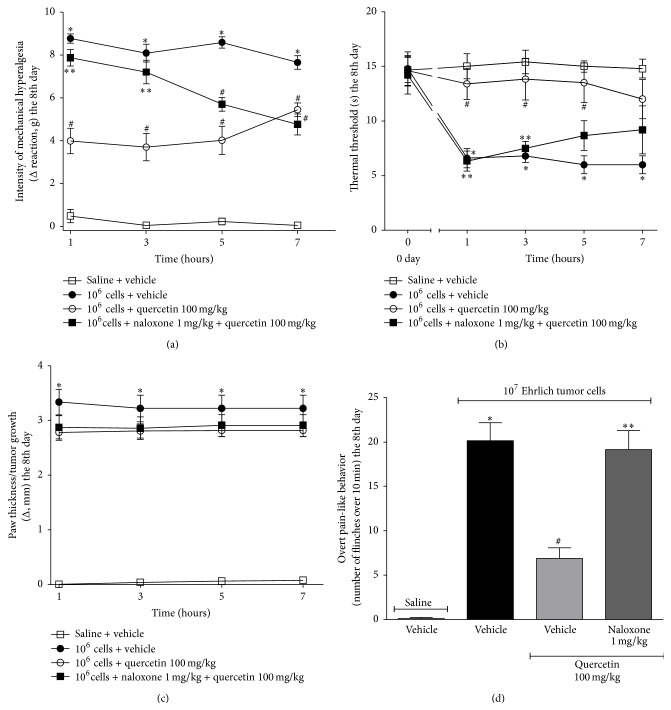
The opioid receptor antagonist, naloxone, inhibits quercetin analgesia in the Ehrlich tumor-induced pain model. Mice were treated with quercetin (100 mg/kg, i.p., starting 10 min after tumor administration) during 8 days after the injection of Ehrlich tumor cells (1 × 10^6^ or 1 × 10^7^ cells/25 *μ*L) or saline and, in the 8th day, one group of mice that received quercetin was also treated with naloxone (1 mg/kg i.p. diluted in saline) 1 h before the treatment with quercetin. The evaluation of mechanical hyperalgesia (a), thermal hyperalgesia (b), and paw thickness (c) was performed 1, 3, 5, and 7 h after the treatments, and the overt pain-like behavior (d) was evaluated 1 h after the treatment. Data are presented as means ± SEM of six mice per group per experiment and representative of two separated experiments: ^*∗*^ *p* < 0.05 compared to the saline group, ^#^ *p* < 0.05 compared to the tumor group, and ^*∗∗*^ *p* < 0.05 compared to the quercetin group. One-way ANOVA followed by Tukey's test.

**Figure 8:**
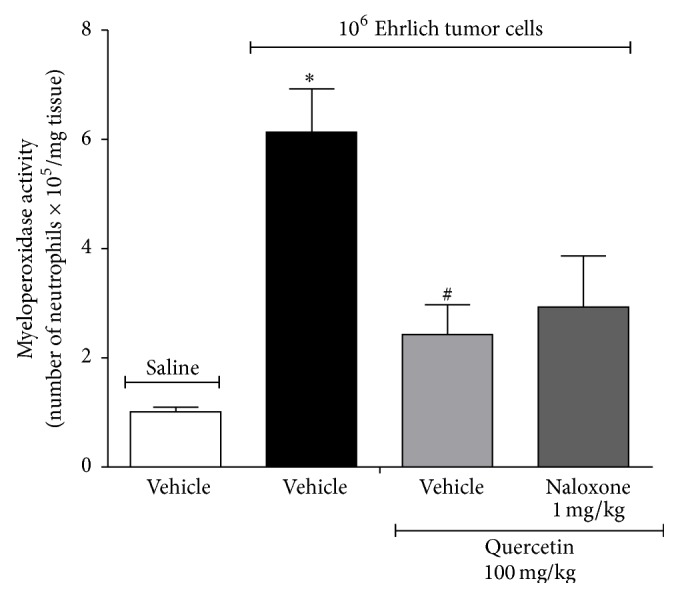
Naloxone did not reverse the effect of quercetin in reducing Ehrlich tumor cells-induced neutrophil recruitment. Mice were treated with quercetin (100 mg/kg, i.p., starting 10 min after tumor administration) during 8 days after the injection of Ehrlich tumor cells (1 × 10^6^ or 1 × 10^7^ cells/25 *μ*L) or saline and, in the 8th day, one group of mice that received quercetin was also treated with naloxone (1 mg/kg i.p. diluted in saline) or its vehicle 1 h before the treatment with quercetin. The neutrophil recruitment was evaluated in samples of paw skin collected after 3 h of the treatment with quercetin by the myeloperoxidase (MPO) activity assay. Data are presented as means ± SEM of six mice per group per experiment and representative of two separated experiments: ^*∗*^ *p* < 0.05 compared to the saline group and ^#^ *p* < 0.05 compared to the tumor group. One-way ANOVA followed by Tukey's test.

**Figure 9:**
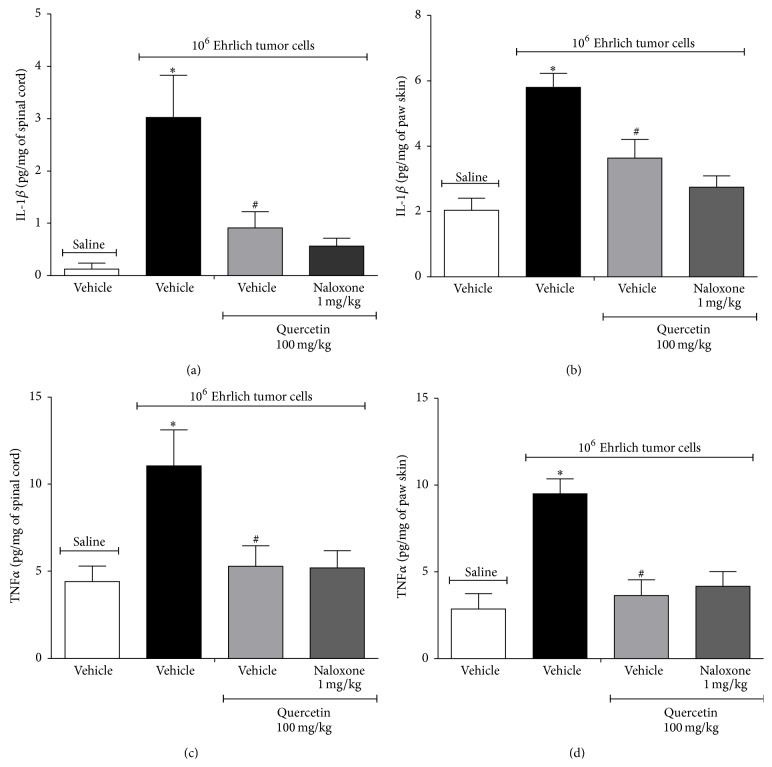
Naloxone did not reverse the effect of quercetin in reducing Ehrlich tumor cells-induced cytokine production. Mice were treated with quercetin (100 mg/kg, i.p., starting 10 min after tumor administration) during 8 days after the injection of Ehrlich tumor cells (1 × 10^6^ or 1 × 10^7^ cells/25 *μ*L) or saline and, in the 8th day, one group of mice that received quercetin was also treated with naloxone (1 mg/kg i.p. diluted in saline) or its vehicle 1 h before the treatment with quercetin. IL-1*β* concentration in spinal cord (a) or paw skin (b) and TNF*α* concentration in spinal cord (c) or paw skin (d) were determined by ELISA 3 h after the treatment with quercetin. Data are presented as means ± SEM of six mice per group per experiment and representative of two separated experiments: ^*∗*^ *p* < 0.05 compared to the saline group and ^#^ *p* < 0.05 compared to the tumor group. One-way ANOVA followed by Tukey's test.

**Figure 10:**
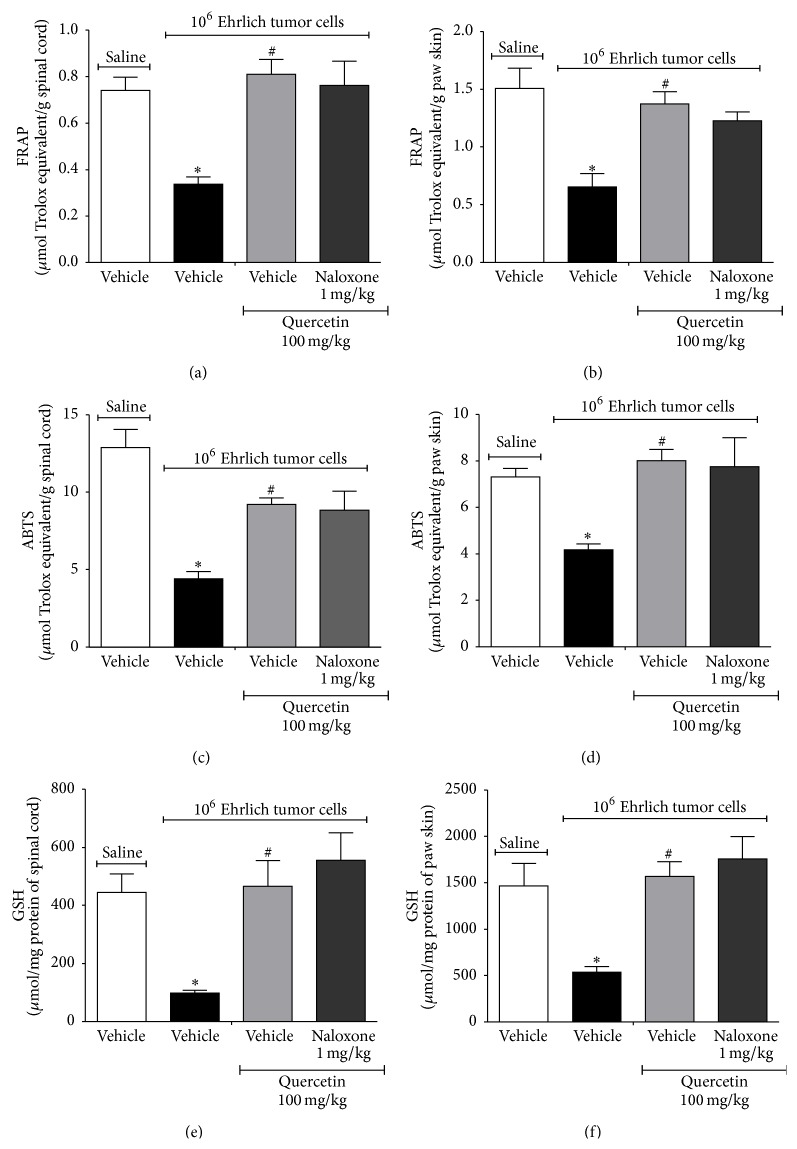
Naloxone did not reverse the effect of quercetin in reducing Ehrlich tumor cells-induced oxidative stress. Mice were treated with quercetin (100 mg/kg, i.p., starting 10 min after tumor administration) during 8 days after the injection of Ehrlich tumor cells (1 × 10^6^ or 1 × 10^7^ cells/25 *μ*L) or saline and, in the 8th day, one group of mice that received quercetin was also treated with naloxone (1 mg/kg i.p. diluted in saline) or its vehicle 1 h before the treatment with quercetin. Three hours after the treatment with quercetin, samples of spinal cord and paw skin were collected for the oxidative stress assays. The FRAP and ABTS ability of spinal cord ((a) and (c), resp.) and paw skin ((b) and (d), resp.) tissues and GSH levels in the spinal cord (e) and paw skin (f) were accessed. Data are presented as means ± SEM of six mice per group per experiment and representative of two separated experiments: ^*∗*^ *p* < 0.05 compared to the saline group and ^#^ *p* < 0.05 compared to the tumor group. One-way ANOVA followed by Tukey's test.

Mice were treated with quercetin as in Figure 2 daily during 8 days. In the 8th day, one group was treated with naloxone (an opioid receptor antagonist, 1 mg/kg, diluted in saline, i.p.) 1 h before the treatment with quercetin (Figure 7) and mechanical hyperalgesia, thermal hyperalgesia, and paw thickness were assessed after 1, 3, 5, and 7 h (Figures 7(a)–7(c)). Quercetin significantly reduced Ehrlich tumor-induced mechanical and thermal hyperalgesia at all time points. The analgesic effect of quercetin was inhibited by naloxone at 1 and 3 h after treatment (Figures 7(a) and 7(b)). As observed in Figure 2, quercetin did not affect paw thickness and naloxone did not alter this absence of effect of quercetin over Ehrlich tumor growth (Figure 7(c)). The same treatment regimen was performed on mice that receive 1 × 10^7^ Ehrlich tumor cells to induce paw flinching. In the 8th day, 1 h after treatment with quercetin, Ehrlich tumor cell-induced paw flinches were evaluated. Quercetin significantly decreased Ehrlich tumor-induced paw flinches and treatment with naloxone inhibited the analgesic effect of quercetin (Figure 7(d)). The dose of naloxone was selected in previous studies [30]. These results indicate that the analgesic effect of quercetin in Ehrlich tumor-induced pain depends on opioid mechanisms. In agreement with our study, the analgesic effect of quercetin in a model of streptozotocin-induced diabetic neuropathic pain [22] and lipopolysaccharide-induced hyperalgesia [59] also depends on opioid mechanisms and is reversible by treatment with naloxone. On the other hand, using the same protocol as for Figure 7, we observed that naloxone did not alter the quercetin inhibition of Ehrlich tumor cells-induced MPO activity in the paw skin (Figure 8). Furthermore, following the same protocol of Figure 7, the effect of naloxone on quercetin inhibition of Ehrlich tumor cells-induced spinal cord and paw skin production of IL-1*β* (Figures 9(a) and 9(b)), TNF*α* (Figures 9(c) and 9(d)), FRAP, ABTS, and GSH (Figures 10(a)–10(f)) were determined. The treatment with naloxone did not alter the anti-inflammatory and antioxidant effects of quercetin (Figures 9 and 10). The anti-inflammatory effect of opioids has already been described. For instance, kappa-opioid agonist exerts anti-inflammatory actions by reduction of adhesion molecule expression, inhibition of cell trafficking, and TNF release and expression [60]. Our data suggest that the analgesic effect of quercetin in Ehrlich tumor-induced cancer pain is dependent on endogenous opioid mechanisms; however, these opioid-dependent mechanisms are not responsible for the anti-inflammatory and antioxidant actions of quercetin observed as reduction of MPO activity, cytokine production, and oxidative stress in the current protocol.

### 3.5. Combined Treatment with Quercetin and Morphine at Doses That Are Ineffective as Single Treatment Reduces Ehrlich Tumor-Induced Pain-Like Responses

**Figure 11:**
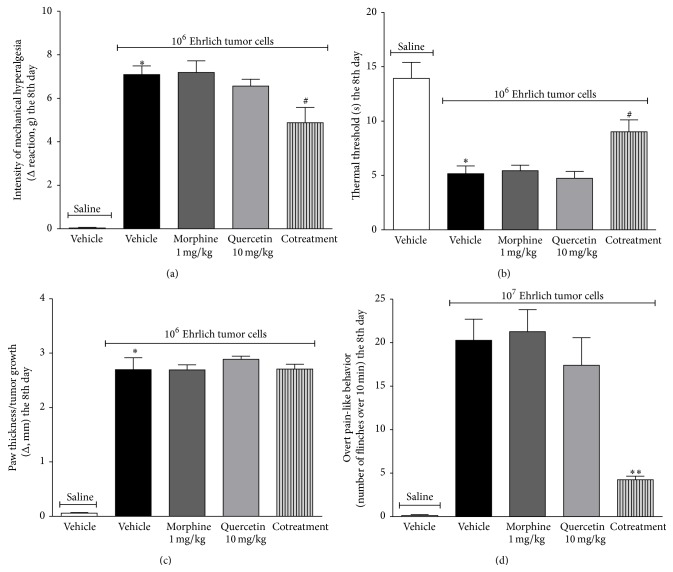
Combined treatment with quercetin and morphine at doses that are ineffective as single treatment reduces Ehrlich tumor-induced pain-like responses. Mice were treated with quercetin (10 mg/kg i.p., a dose without significant analgesic effect* per se*), before the injection of Ehrlich tumor cells (1 × 10^6^ or 1 × 10^7^ cells, i.pl.). Mice were treated daily during 8 days and, in the 8th day, mice were treated with quercetin and after 2 h and 15 min received morphine (1 mg/kg i.p., a dose without significant analgesic effect* per se*). Mechanical (a) and thermal hyperalgesia (b), paw thickness (c), and overt pain-like behavior (d) were evaluated 3 h after the last quercetin treatment. Data are presented as means ± SEM of six mice per group per experiment and representative of two separated experiments: ^*∗*^ *p* < 0.05 compared to the saline group, ^#^ *p* < 0.05 compared to the tumor group, and ^*∗∗*^ *p* < 0.05 compared to the quercetin 10 mg/kg and morphine 1 mg/kg. One-way ANOVA followed by Tukey's test.

Mice were treated with quercetin (10 mg/kg i.p., a dose without significant analgesic effect* per se*, Figure 2) 10 min after administration of Ehrlich tumor cells (1 × 10^6^ or 1 × 10^7^ cells, i.pl.). Mice were treated daily during 8 days. In the 8th day, mice were treated with morphine (1 mg/kg i.p., a dose without significant analgesic effect* per se*) 2 h and 15 min after quercetin administration. Mechanical hyperalgesia, thermal hyperalgesia, paw thickness (1 × 10^6^ Ehrlich tumor cells), and paw flinching (1 × 10^7^ Ehrlich tumor cells) were assessed 45 min after morphine treatment or 3 h after quercetin treatment (Figures 11(a)–11(d)). Ehrlich tumor-induced mechanical and thermal hyperalgesia were not reduced by treatment with quercetin (10 mg/kg, i.p.) or morphine (1 mg/kg, i.p.) alone. However, the cotreatment with quercetin and morphine significantly reduced the mechanical (Figure 11(a)) and thermal hyperalgesia (Figure 11(b)). Ehrlich tumor-induced increase in the paw thickness was not altered by quercetin, morphine, or cotreatment with both molecules (Figure 11(c)). Finally, Ehrlich tumor-induced paw flinches were also reduced by cotreatment with quercetin and morphine, but not by quercetin or morphine alone (Figure 11(d)). These results suggest a synergic analgesic effect of quercetin and morphine over Ehrlich tumor-induced pain. Moreover, this synergy was more evident in the overt pain-like response, which clearly showed a potentiation of analgesia (Figure 11(d)). Therefore, these results on synergy or even potentiation of analgesia by cotreatment with quercetin and morphine at doses without analgesic effect as single treatment are important in the sense that indicates possible reduction of morphine dosage by combination with quercetin treatment to control cancer pain.

Evidence supports a synergy/potentiation between quercetin and opioids/morphine in other models, indicating that this effect should be addressed. For instance, quercetin reduces the morphine tolerance [61], reduces naloxone-precipitated withdrawal contracture of the acute morphine-dependent guinea-pig ileum [62], and exhibits morphine-like inhibition of acetylcholine release in the coaxially stimulated ileum [63]. Therefore, the opioid-related actions of quercetin are consistent in varied systems and may contribute to reduce morphine dosage ([22, 61] and present data) as well as morphine tolerance [62]. Mechanistically, quercetin inhibits morphine tolerance by inhibiting nitric oxide synthase activity [61]. Therefore, it is likely that quercetin potentiates opioid activity indirectly by inhibiting mechanisms that would limit opioid effects and not by inducing opioid release or binding to and activating opioid receptors, which explain a synergic/potentiating effect of quercetin and morphine.

In addition to the analgesic effects, opioids also present anti-inflammatory actions* in vitro* and* in vivo* [64, 65]. The present results suggest that quercetin inhibits Ehrlich tumor cells-induced pain by two independent mechanisms: (a) an opioid-related analgesic mechanism and (b) an anti-inflammatory/antioxidant mechanism. The opioid-related mechanism might present central analgesic effects since per oral treatment with quercetin inhibited diabetic neuropathic pain in mice in the tail-immersion in warm water test, which evaluates the involvement of central nociceptive responses, in a naloxone sensitive manner [22]. The anti-inflammatory/antioxidant mechanism of quercetin is related to the inhibition of proinflammatory signaling pathways and intrinsic structural antioxidant chemical groups of quercetin [23].

In conclusion, the present study demonstrates that quercetin inhibits Ehrlich tumor-induced pain by mechanisms targeting peripheral and spinal cord oxidative stress and hyperalgesic cytokine production as well as inducing an opioid-related analgesic mechanism, resulting in potentiation of morphine analgesia. The analgesic dose of quercetin did not alter tumor growth demonstrating; therefore, its analgesia does not depend on reducing tumor mass.
